# Comparative study of radiation dose between tomosynthesis and standard compression views in mammography

**DOI:** 10.1186/bcr3777

**Published:** 2015-11-05

**Authors:** Alice Bannister, Julie Scudder

**Affiliations:** 1 Guys and St Thomas' NHS Foundation Trust, London, UK

## Introduction

Objectives were a comparative study of the radiation dose of two-view digital breast tomosynthesis (DBT) and two-view spot compression views in a symptomatic breast service.

## Methods

Two hundred patients were included in the study, 100 who had undergone two-view spot compression views and 100 two-view DBT. DBT was carried out using GE Seno Claire with an iso-dose setting and grid system. A retrospective computer-based search of patients in the two categories was undertaken and the accumulative dose for each technique was identified and recorded, as was the thickness of the breast from the original cc mammogram projection. The percentage variance of dose between DBT and spot compression views was calculated according to breast thickness.

## Results

The mean accumulative glandular dose for the whole group regardless of breast thickness was 2.84 for DBT compared with 3.50 for spot compression views. In this patient population, the AGD was lower for DBT than for FFDM in 64 % of the patients. When patients were categorized according to breast thickness, the accumulative glandular dose of DBT was on average 22 % less than spot compression mammography with a reduction ranging from 53 to 1 %. There was no evidence in this study that dose reduction with DBT significantly increased with increasing breast thickness.

## Conclusion

The radiation dose of patients undergoing two-view DBT on average showed a significant reduction compared to two-view spot compression views. The dose reduction may be attributed to the grid and iso-dose technology used in the selected DBT system.

